# Supplementary material for: DNA replication machinery prevents Rad52-dependent single-strand annealing that leads to gross chromosomal rearrangements at centromeres
Source: Commun Biol. 2020 Apr 30;3:202. doi: 10.1038/s42003-020-0934-0 (PMC7193609; doi:10.1038/s42003-020-0934-0)
Supplement: Supplementary file 1 — Supplementary Information [file 42003_2020_934_MOESM1_ESM.pdf]

Supplementary Figures

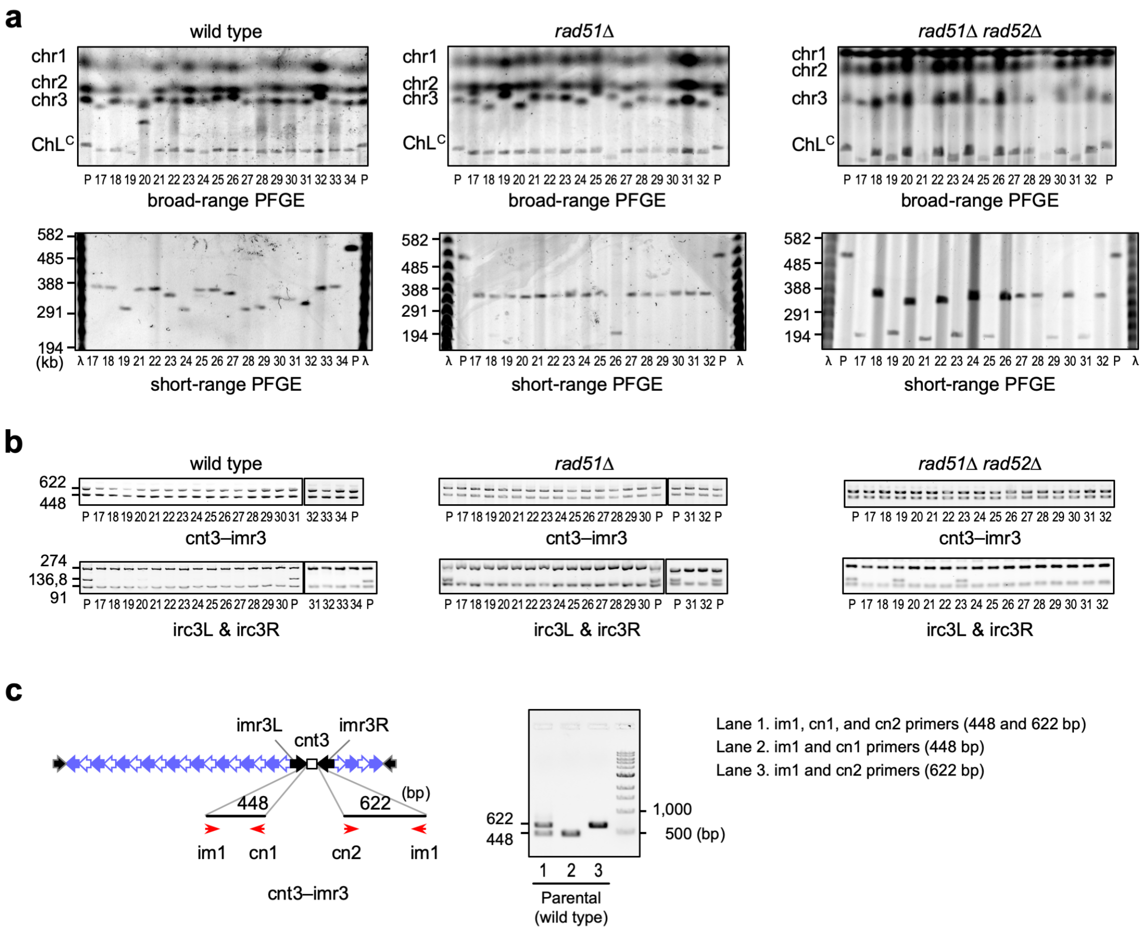

Supplementary Fig. 1 GCR products formed in the wild-type, *rad51Δ*, and *rad51Δ rad52Δ* strains. **a** Chromosomal DNAs from parental and independent GCR clones of the wild-type, *rad51Δ*, and *rad51Δ rad52Δ* strains (TNF5369, 5411, and 7553, respectively) were separated by broad- and short-range PFGE and stained with EtBr. **b** PCR analysis of GCR products. Both sides of the cnt3–imr3 junctions (cnt3–imr3) and outermost repeats (irc3L & irc3R) were examined. **c** Separate amplification of the left and right sides of the cnt3-imr3 junctions. The parental ChL^{C} of the wild-type strain was amplified using the indicated three different sets of primers. The lengths of DNA fragments in the 1 kb DNA Ladder (New England Biolabs, N3232S) are shown on the right of the image. Uncropped images of the gels presented here are shown in Supplementary Fig. 10

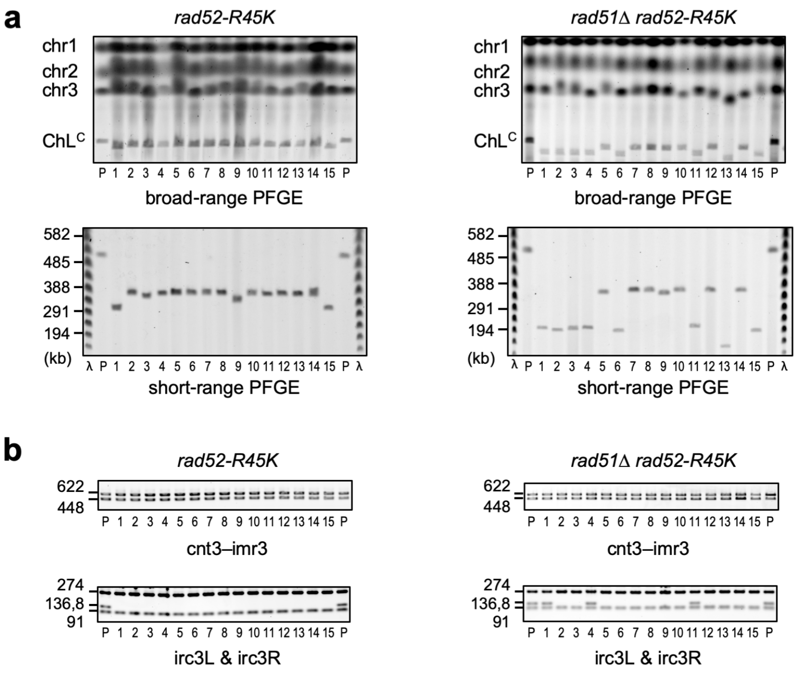

Supplementary Fig. 2 GCR products formed in the *rad52-R45K* and *rad51Δ rad52-R45K* strains. **a** Chromosomal DNAs from parental and independent GCR clones of the *rad52-R45K* and *rad51Δ rad52-R45K* strains (TNF6599 and 6616, respectively) were separated by broad- and short-range PFGE and stained with EtBr. **b** PCR analysis of GCR products. Both sides of the cnt3–imr3 junctions (cnt3–imr3) and outermost repeats (irc3L & irc3R) were examined. Uncropped images of the gels presented here are shown in Supplementary Fig. 11

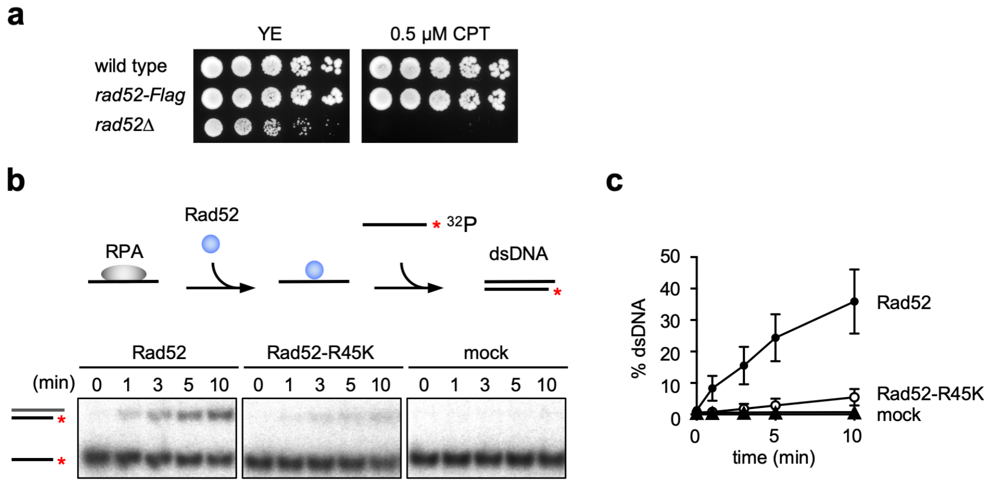

Supplementary Fig. 3 The *rad52-R45K* mutation impairs the SSA activity in the presence of RPA. **a** Exponentially growing cells of the wild-type, *rad52-Flag*, and *rad52Δ* strains (TNF35, 2134, and 3696, respectively) were 5-fold serially diluted in water and spotted onto the YE plates supplemented with 0.5 μM CPT. **b** SSA assays were performed as in Fig. 4, except that Oligo508 was pre-incubated at 30°C for 5 min in the presence of 0.4 nM RPA. **c** Percentages of dsDNA signals in whole-lane signals. The mean and s.d. of three independent experiments. Uncropped images of the gels presented in **b** are shown in Supplementary Fig. 12. Source data for the graphs in **c** are available in Supplementary Data 1

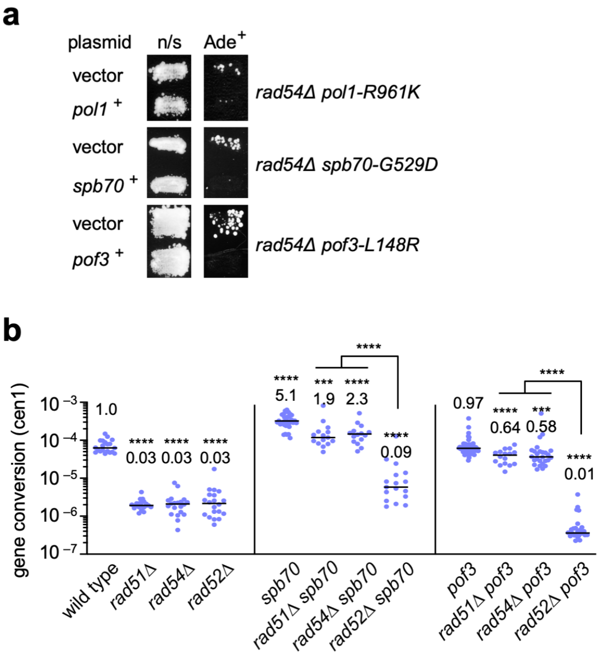

Supplementary Fig. 4 Mutations in *pol1*, *spb70*, and *pof3* mutations increase Rad51/Rad54–independent but Rad52-dependent recombination at centromeres. **a** Complementation assays. An empty plasmid or a plasmid containing the corresponding wild-type gene was introduced into the *rad54*Δ *pol1-R961K*, *rad54*Δ *spb70-G529D*, and *rad54*Δ *pof3-L148R* strains (TNF4125, 4109, and 4102, respectively). Transformants grown on non-selective EMM+A (n/s) were transferred to EMM+G plates, on which only Ade^{+} recombinants can grow. Cells were incubated at 33°C. **b** Gene conversion rates at cen1 in the wild-type, *rad51*Δ, *rad54*Δ, *rad52*Δ, *spb70-G529D*, *rad51*Δ *spb70-G529D*, *rad54*Δ *spb70-G529D*, *rad52*Δ *spb70-G529D*, *pof3-L148R*, *rad51*Δ *pof3-L148R*, *rad54*Δ *pof3-L148R*, and *rad52*Δ *pof3-L148R* strains (TNF3347, 3446, 3452, 3459, 4174, 4544, 4196, 4609, 4085, 4099, 4119, and 4111, respectively). In contrast to *pol1-R961K* and *pof3-L148R*, the *spb70-G529D* mutation increased gene conversion in the wild-type background. *** $P < 0.001$; **** $P < 0.0001$. Source data for the graphs in **b** are available in Supplementary Data 1

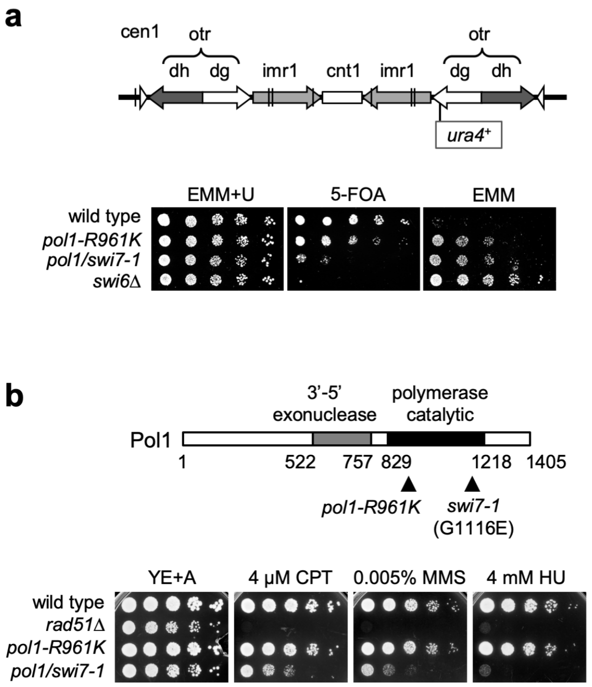

Supplementary Fig. 5 The *pol1-R916K* mutation partially impairs centromere gene silencing but does not affects sensitivity to DNA damaging agents. **a** Transcriptional silencing of the *ura4*$^{+}$ gene integrated at cen1. Exponentially growing cells of the wild-type, *pol1-R961K*, *pol1/swi7-1*, and *swi6*Δ (TNF2648, 4208, 4625, and 3220, respectively) in EMM+U were 5-fold serially diluted in water and spotted onto the indicated plates. Cells were grown at 30°C. **b** Exponentially growing cells of the wild-type, *rad51*Δ, *pol1-R961K*, and *pol1/swi7-1* (TNF3347, 3446, 4235, and 4328, respectively) in YE3S were 5-fold serially diluted and spotted onto YE+A supplemented with the indicated concentrations CPT, MMS, or HU. Positions of *pol1-R961K* and *pol1/swi7-1* mutation sites in Pol1 are indicated on the top of panels

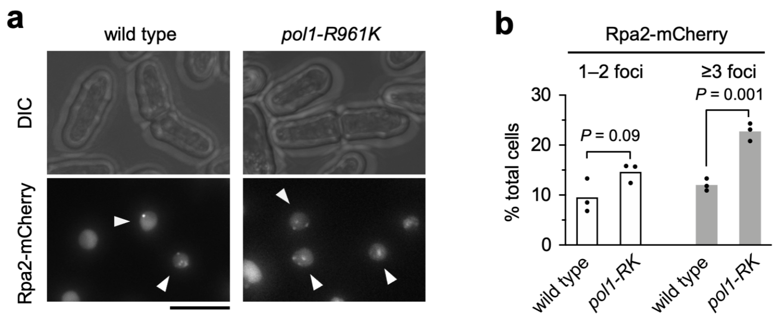

Supplementary Fig. 6 Spontaneous focus formation of Rpa2-mCherry. Focus formation of mCherry-tagged Rpa2, a subunit of the RPA complex that binds ssDNA. Rpa2-mCherry was expressed under the native *rpa2* promoter at the original chromosomal locus. **a** Exponentially growing cells in EMM at 30°C were collected and observed using a DeltaVision Personal fluorescence microscopy system (GE Healthcare Life Science). Scale bar indicates 5 μm. DIC, differential interference contrast. Arrow heads indicate nuclei containing Rpa2-mCherry foci. **b** Percentages of cells containing 1–2 and ≥3 Rpa2-mCherry foci in the wild-type and *po1-R961K* strains (TNF5492 and 5511, respectively). Over 400 cells were analysed in each measurement. Bars represent the mean of three independent experiments (dots). Statistical analysis was performed by two-tailed Student's *t*-test. Source data for the graphs in **b** are available in Supplementary Data 1

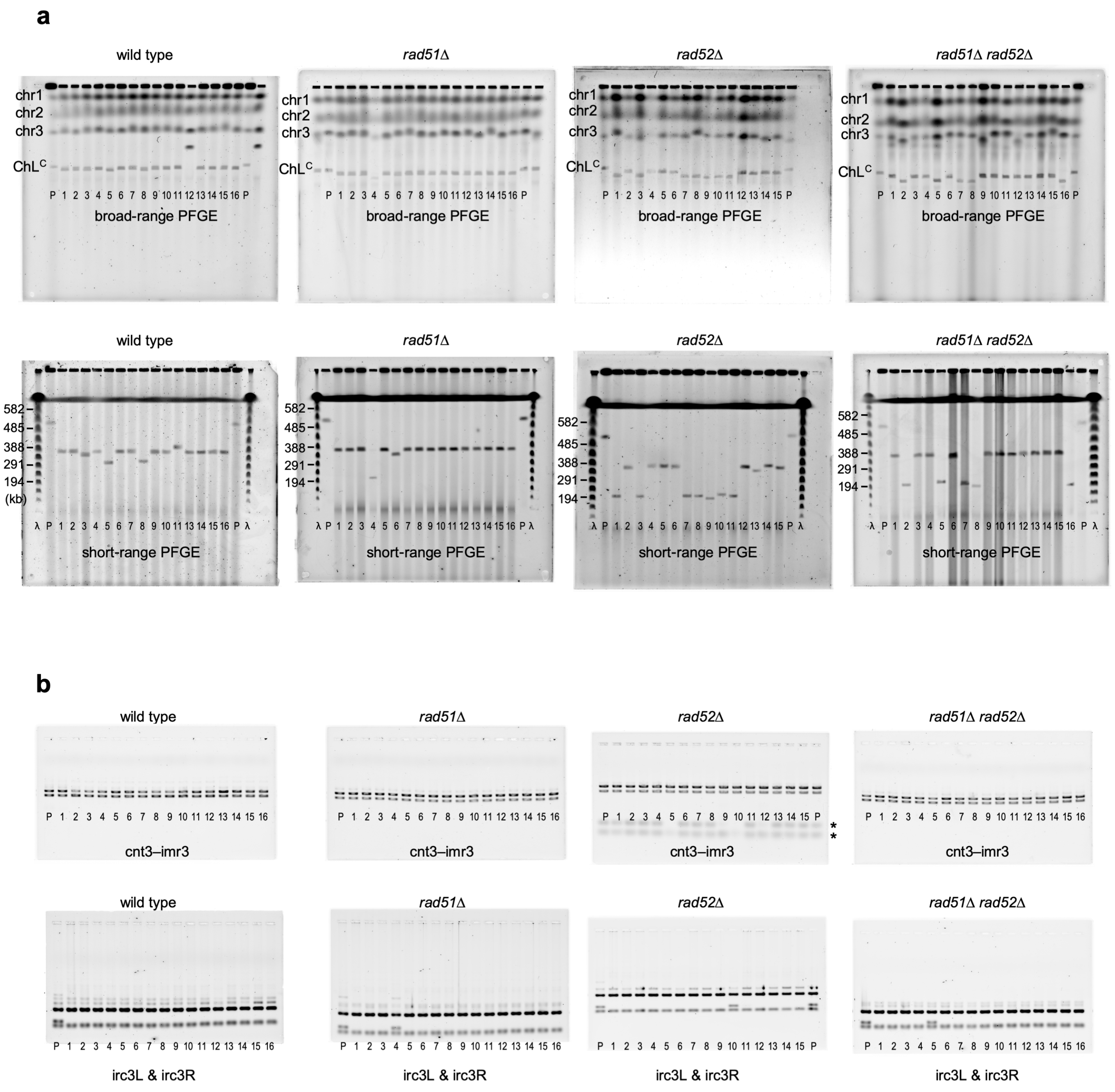

Supplementary Fig. 7 Uncropped images of the gels presented in Figs. 2b and 2c. **a** PFGE images. **b** Standard agarose gel images. Asterisks indicate DNA bands that have been loaded onto the gel previously

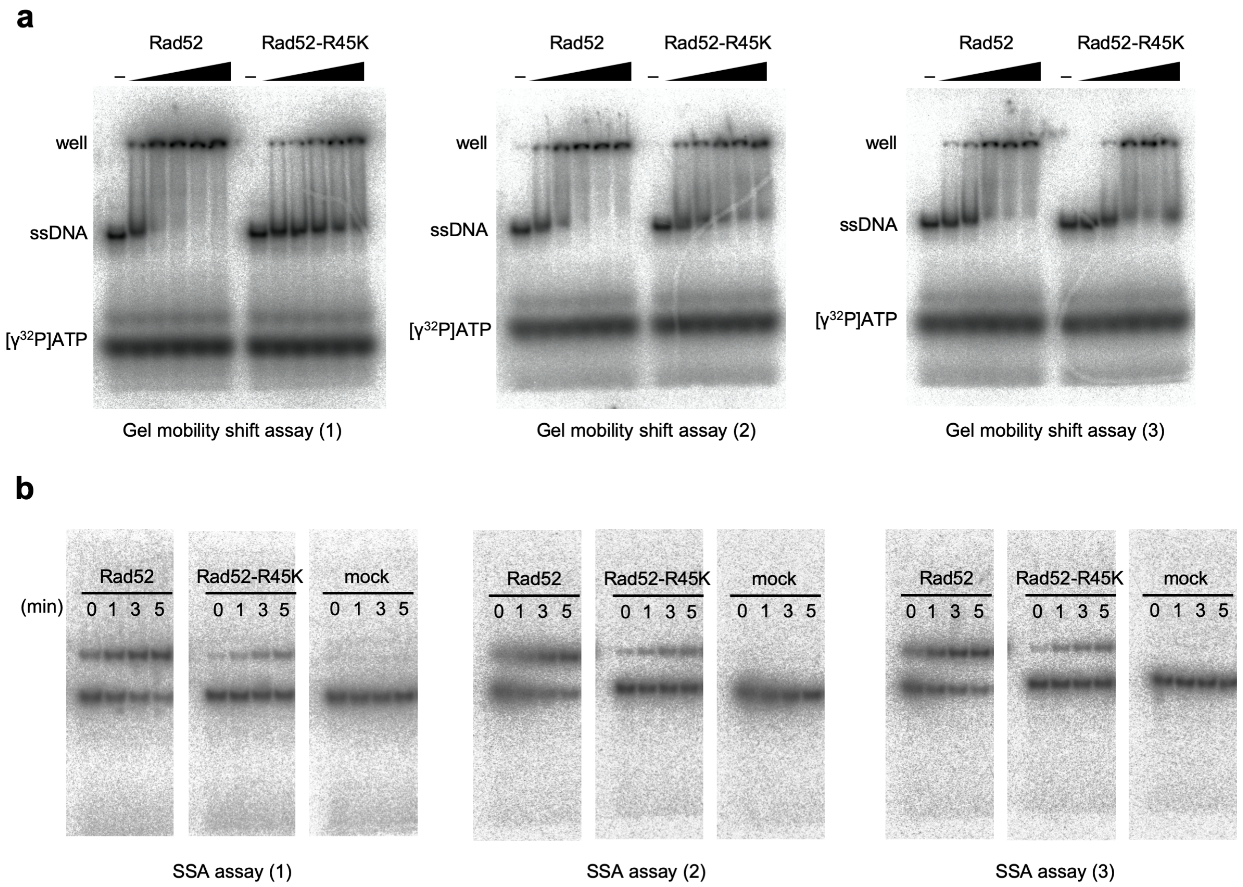

Supplementary Fig. 8 Uncropped images of the gels presented in Figs. 4b and 4e. **a** Gel mobility shift assays. **b** SSA assays

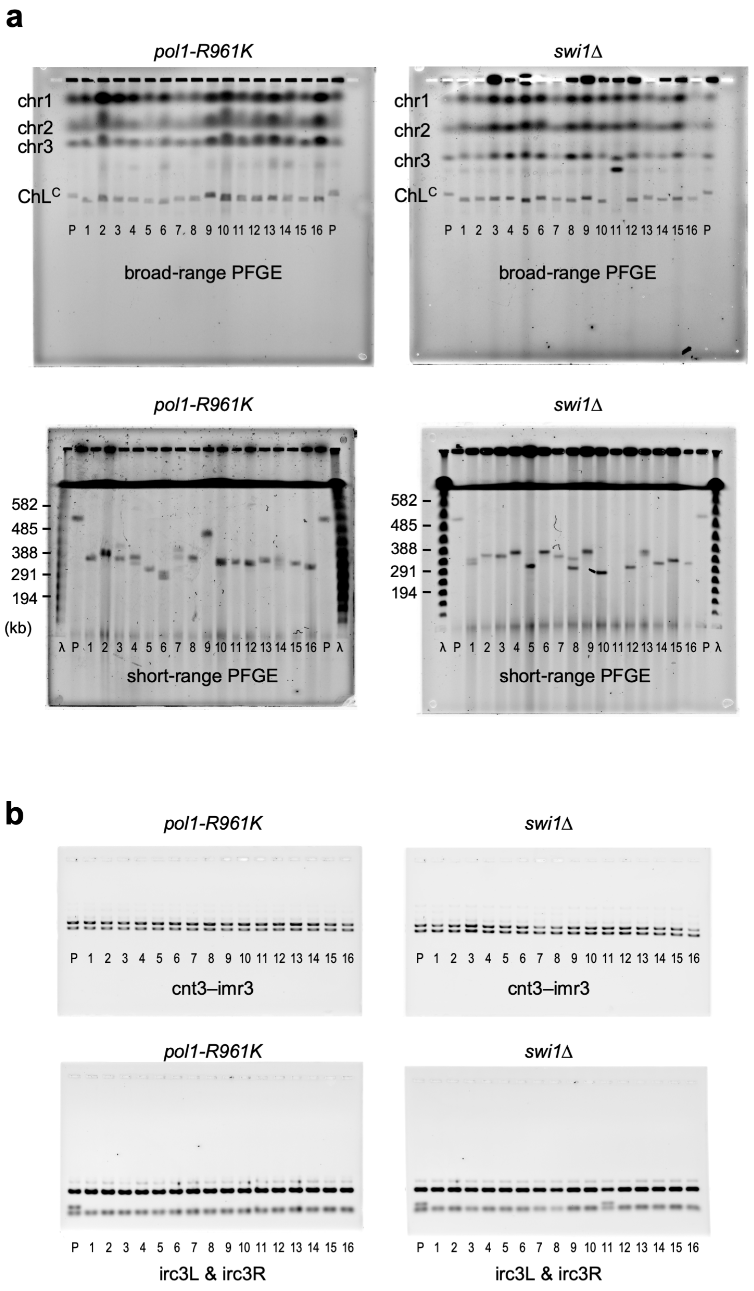

Supplementary Fig. 9 Uncropped images of gels presented in Figs. 7c and 7d. **a** PFGE images. **b** Standard agarose gel images

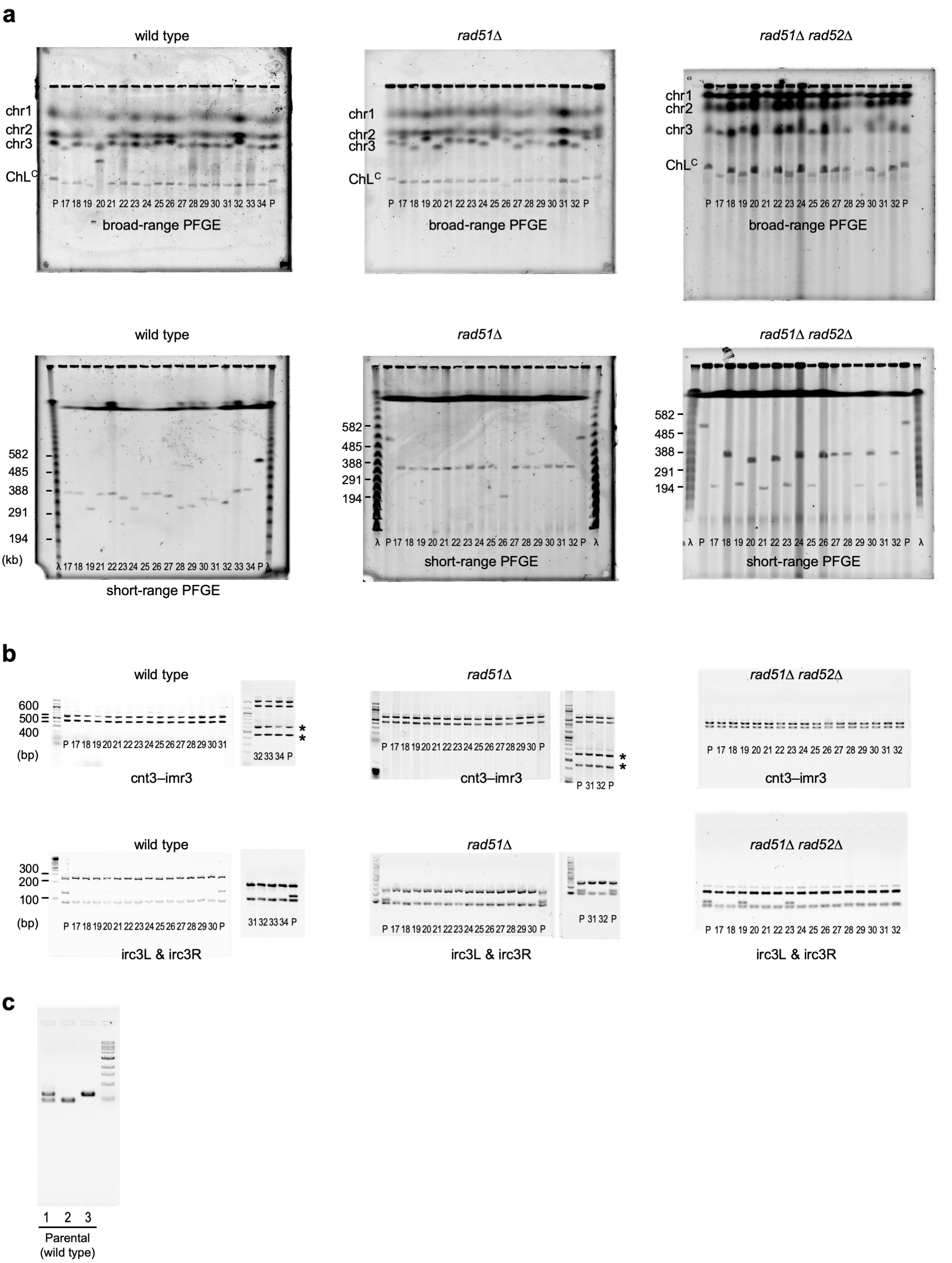

Supplementary Fig. 10 Uncropped images of the gels presented in Supplementary Fig. 1. **a** PFGE images. **b** and **c** Standard agarose gel images. Asterisks indicate DNA bands that have been loaded onto the gel previously

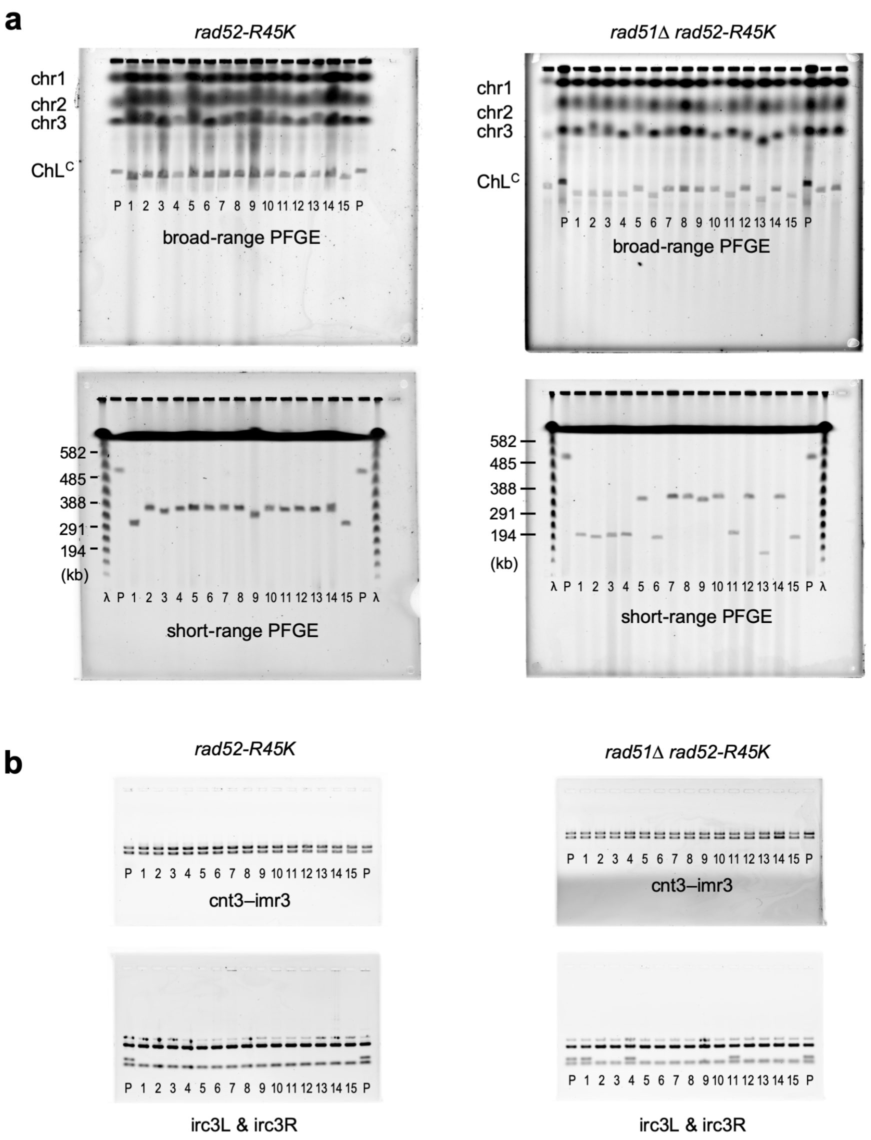

Supplementary Fig. 11 Uncropped images of the gels presented in Supplementary Fig. 2. **a** PFGE images. **b** Standard agarose gel images

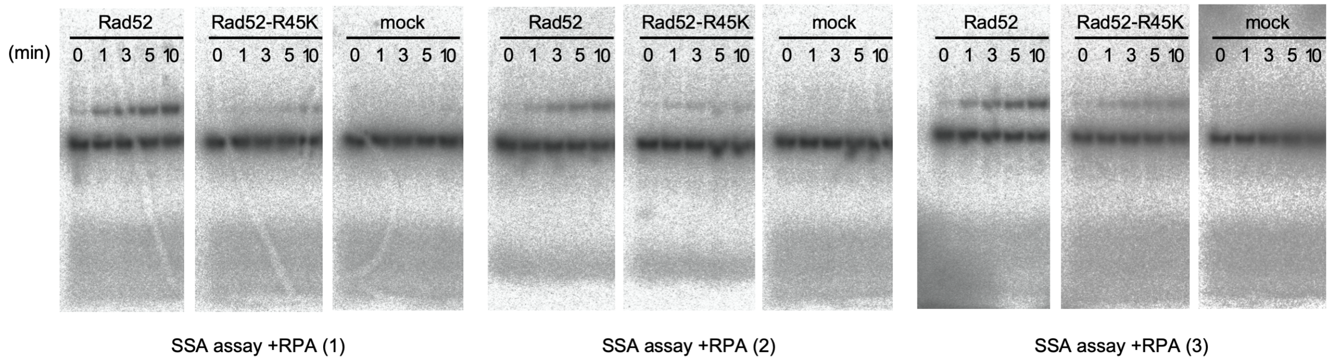

Supplementary Fig. 12 Uncropped images of the gels presented in Supplementary Fig. 3b

Supplementary Tables

Supplementary Table 1. The yeast strains used in this study.

Strain	Genotype
TNF5369	*h⁻, smt0, ade6Δ-D, ura4-D18, leu1-32,* ChL^C [*ubc11::LEU2$^+$, cwf20::ura4$^+$, ade6$^+$*]
TNF5411	*h⁻, smt0, ade6Δ-D, ura4-D18, leu1-32,* ChL^C [*ubc11::LEU2$^+$, cwf20::ura4$^+$, ade6$^+$*], *rad51::natMX6*
TNF7493	*h⁻, smt0, ade6Δ-D, ura4-D18, leu1-32,* ChL^C [*ubc11::LEU2$^+$, cwf20::ura4$^+$, ade6$^+$*], *rad52::kanMX6*
TNF7553	*h⁻, smt0, ade6Δ-D, ura4-D18, leu1-32,* ChL^C [*ubc11::LEU2$^+$, cwf20::ura4$^+$, ade6$^+$*], *rad51::natMX6, rad52::kanMX6*
TNF6599	*h⁻, smt0, ade6Δ-D, ura4-D18, leu1-32,* ChL^C [*ubc11::LEU2$^+$, cwf20::ura4$^+$, ade6$^+$*], *rad52-R45K*
TNF6707	*h⁻, smt0, ade6Δ-D, ura4-D18, leu1-32,* ChL^C [*ubc11::LEU2$^+$, cwf20::ura4$^+$, ade6$^+$*], *rti1::hphMX6*
TNF7879	*h⁻, smt0, ade6Δ-D, ura4-D18, leu1-32,* ChL^C [*ubc11::LEU2$^+$, cwf20::ura4$^+$, ade6$^+$*], *rad52-R45K, rti1::hphMX6*
TNF6616	*h⁻, smt0, ade6Δ-D, ura4-D18, leu1-32,* ChL^C [*ubc11::LEU2$^+$, cwf20::ura4$^+$, ade6$^+$*], *rad51::natMX6, rad52-R45K*
TNF6725	*h⁻, smt0, ade6Δ-D, ura4-D18, leu1-32,* ChL^C [*ubc11::LEU2$^+$, cwf20::ura4$^+$, ade6$^+$*], *rad51::natMX6, rti1::hphMX6*
TNF7886	*h⁻, smt0, ade6Δ-D, ura4-D18, leu1-32,* ChL^C [*ubc11::LEU2$^+$, cwf20::ura4$^+$, ade6$^+$*], *rad51::natMX6, rad52-R45K*, *rti1::hphMX6*
TNF6618	*h⁻, smt0, ade6Δ-D, ura4-D18, leu1-32,* ChL^C [*ubc11::LEU2$^+$, cwf20::ura4$^+$, ade6$^+$*], *msh2::hphMX6*
TNF6867	*h⁻, smt0, ade6Δ-D, ura4-D18, leu1-32,* ChL^C [*ubc11::LEU2$^+$, cwf20::ura4$^+$, ade6$^+$*], *msh3::hphMX6*
TNF6869	*h⁻, smt0, ade6Δ-D, ura4-D18, leu1-32,* ChL^C [*ubc11::LEU2$^+$, cwf20::ura4$^+$, ade6$^+$*], *msh6::hphMX6*
TNF6620	*h⁻, smt0, ade6Δ-D, ura4-D18, leu1-32,* ChL^C [*ubc11::LEU2$^+$, cwf20::ura4$^+$, ade6$^+$*], *mlh1::hphMX6*
TNF6627	*h⁻, smt0, ade6Δ-D, ura4-D18, leu1-32,* ChL^C [*ubc11::LEU2$^+$, cwf20::ura4$^+$, ade6$^+$*], *rad52-R45K, msh2::hphMX6*
TNF6649	*h⁻, smt0, ade6Δ-D, ura4-D18, leu1-32,* ChL^C [*ubc11::LEU2$^+$, cwf20::ura4$^+$, ade6$^+$*], *rad51::natMX6, msh2::hphMX6*
TNF7081	*h⁻, smt0, ade6Δ-D, ura4-D18, leu1-32,* ChL^C [*ubc11::LEU2$^+$, cwf20::ura4$^+$, ade6$^+$*], *rad51::natMX6, msh3::hphMX6*
TNF6908	*h⁻, smt0, ade6Δ-D, ura4-D18, leu1-32,* ChL^C [*ubc11::LEU2$^+$, cwf20::ura4$^+$, ade6$^+$*], *rad51::natMX6, msh6::hphMX6*

TNF6651	*h*$^-$*, smt0, ade6Δ-D, ura4-D18, leu1-32,* ChL^C [*ubc11::LEU2*$^+$*, cwf20::ura4*$^+$*, ade6*$^+$], *rad51::natMX6, mlh1::hphMX6*
TNF6697	*h*$^-$*, smt0, ade6Δ-D, ura4-D18, leu1-32,* ChL^C [*ubc11::LEU2*$^+$*, cwf20::ura4*$^+$*, ade6*$^+$], *rad51::natMX6, rad52-R45K, msh2::hphMX6*
TNF5669	*h*$^-$*, smt0, ade6Δ-D, ura4-D18, leu1-32, ChLC [ubc11::LEU2+, cwf20::ura4+, ade6+], mus81::hphMX6*
TNF6614	*h*$^-$*, smt0, ade6Δ-D, ura4-D18, leu1-32, ChLC [ubc11::LEU2+, cwf20::ura4+, ade6+], rad52-R45K, mus81::hphMX6*
TNF5974	*h*$^-$*, smt0, ade6Δ-D, ura4-D18, leu1-32, ChLC [ubc11::LEU2+, cwf20::ura4+, ade6+], rad51::natMX6, mus81::hphMX6*
TNF6648	*h*$^-$*, smt0, ade6Δ-D, ura4-D18, leu1-32, ChLC [ubc11::LEU2+, cwf20::ura4+, ade6+], rad51::natMX6, rad52-R45K, mus81::hphMX6*
TNF6678	*h*$^-$*, smt0, ade6Δ-D, ura4-D18, leu1-32,* ChL^C [*ubc11::LEU2*$^+$*, cwf20::ura4*$^+$*, ade6*$^+$], *pol1-R961K*
TNF6952	*h*$^-$*, smt0, ade6Δ-D, ura4-D18, leu1-32,* ChL^C [*ubc11::LEU2*$^+$*, cwf20::ura4*$^+$*, ade6*$^+$], *swi1::hphMX6*
TNF6833	*h*$^-$*, smt0, ade6Δ-D, ura4-D18, leu1-32,* ChL^C [*ubc11::LEU2*$^+$*, cwf20::ura4*$^+$*, ade6*$^+$], *rad51::natMX6, pol1-R961K*
TNF7909	*h*$^-$*, smt0, ade6Δ-D, ura4-D18, leu1-32,* ChL^C [*ubc11::LEU2*$^+$*, cwf20::ura4*$^+$*, ade6*$^+$], *rad51::natMX6, swi1::hphMX6*
TNF6695	*h*$^-$*, smt0, ade6Δ-D, ura4-D18, leu1-32,* ChL^C [*ubc11::LEU2*$^+$*, cwf20::ura4*$^+$*, ade6*$^+$], *rad52-R45K, pol1-R961K*
TNF6954	*h*$^-$*, smt0, ade6Δ-D, ura4-D18, leu1-32,* ChL^C [*ubc11::LEU2*$^+$*, cwf20::ura4*$^+$*, ade6*$^+$], *rad52-R45K, swi1::hphMX6*
TNF3631	*h*$^+$*, ade6Δ-D, ura4::ade6B(R)-cen1(Sn-Sn)-ade6X(L)*
TNF3635	*h*$^+$*, ade6Δ-D, ura4::ade6B(R)-cen1(Sn-Sn)-ade6X(L), rad51::kanMX6*
TNF5995	*h*$^+$*, ade6Δ-D, ura4::ade6B(R)-cen1(Sn-Sn)-ade6X(L), rad52-R45K*
TNF5389	*h*$^+$*, ade6Δ-D, ura4::ade6B(R)-cen1(Sn-Sn)-ade6X(L), rti1::hphMX6*
TNF7878	*h*$^+$*, ade6Δ-D, ura4::ade6B(R)-cen1(Sn-Sn)-ade6X(L), rad52-R45K, rti1::hphMX6*
TNF6021	*h*$^+$*, ade6Δ-D, ura4::ade6B(R)-cen1(Sn-Sn)-ade6X(L), rad51::kanMX6, rad52-R45K*
TNF5427	*h*$^+$*, ade6Δ-D, ura4::ade6B(R)-cen1(Sn-Sn)-ade6X(L), rad51::kanMX6, rti1::hphMX6*
TNF7890	*h*$^+$*, ade6Δ-D, ura4::ade6B(R)-cen1(Sn-Sn)-ade6X(L), rad51::kanMX6, rad52-R45K, rti1::hphMX6*
TNF3643	*h*$^+$*, ade6Δ-D, ura4::ade6B(R)-cen1(Sn-Sn)-ade6X(L), rad52::kanMX6*
TNF6128	*h*$^+$*, ade6Δ-D, ura4::ade6B(R)-cen1(Sn-Sn)-ade6X(L), msh2::hphMX6*
TNF6518	*h*$^+$*, ade6Δ-D, ura4::ade6B(R)-cen1(Sn-Sn)-ade6X(L), mus81::hphMX6*

TNF6136	*h^+, ade6Δ-D, ura4::ade6B(R)-cen1(Sn-Sn)-ade6X(L), rad51::kanMX6, msh2::hphMX6*
TNF6569	*h^+, ade6Δ-D, ura4::ade6B(R)-cen1(Sn-Sn)-ade6X(L), rad51::kanMX6, mus81::hphMX6*
TNF3645	*h^+, ade6Δ-D, ura4::ade6B(R)-cen1(Sn-Sn)-ade6X(L), rad54::kanMX6*
TNF3643	*h^+, ade6Δ-D, ura4::ade6B(R)-cen1(Sn-Sn)-ade6X(L), rad52::kanMX6*
TNF4215	*h^+, ade6Δ-D, ura4::ade6B(R)-cen1(Sn-Sn)-ade6X(L), pol1-R961K*
TNF4371	*h^+, ade6Δ-D, ura4::ade6B(R)-cen1(Sn-Sn)-ade6X(L), rad51::kanMX6, pol1-R961K*
TNF4378	*h^+, ade6Δ-D, ura4::ade6B(R)-cen1(Sn-Sn)-ade6X(L), rad54::kanMX6, pol1-R961K*
TNF4350	*h^+, ade6Δ-D, ura4::ade6B(R)-cen1(Sn-Sn)-ade6X(L), rad52::kanMX6, pol1-R961K*
TNF3347	*h^+, ade6Δ-D, imr1L(Sn:ade6B), imr1R(Sn:ade6X)*
TNF3446	*h^+, ade6Δ-D, imr1L(Sn:ade6B), imr1R(Sn:ade6X), rad51::kanMX6*
TNF3452	*h^+, ade6Δ-D, imr1L(Sn:ade6B), imr1R(Sn:ade6X), rad54::kanMX6*
TNF3459	*h^+, ade6Δ-D, imr1L(Sn:ade6B), imr1R(Sn:ade6X), rad52::kanMX6*
TNF4235	*h^+, ade6Δ-D, imr1L(Sn:ade6B), imr1R(Sn:ade6X), pol1-R961K*
TNF4300	*h^+, ade6Δ-D, imr1L(Sn:ade6B), imr1R(Sn:ade6X), rad51::kanMX6, pol1-R961K*
TNF4252	*h^+, ade6Δ-D, imr1L(Sn:ade6B), imr1R(Sn:ade6X), rad54::kanMX6, pol1-R961K*
TNF4253	*h^+, ade6Δ-D, imr1L(Sn:ade6B), imr1R(Sn:ade6X), rad52::kanMX6, pol1-R961K*
TNF4174	*h^+, ade6Δ-D, imr1L(Sn:ade6B), imr1R(Sn:ade6X), spb70-G529D*
TNF4544	*h^+, ade6Δ-D, imr1L(Sn:ade6B), imr1R(Sn:ade6X), rad51::kanMX6, spb70-G529D*
TNF4196	*h^+, ade6Δ-D, imr1L(Sn:ade6B), imr1R(Sn:ade6X), rad54::kanMX6, spb70-G529D*
TNF4609	*h^+, ade6Δ-D, imr1L(Sn:ade6B), imr1R(Sn:ade6X), rad52::kanMX6, spb70-G529D*
TNF4085	*h^+, ade6Δ-D, imr1L(Sn:ade6B), imr1R(Sn:ade6X), pof3-L148R*
TNF4099	*h^+, ade6Δ-D, imr1L(Sn:ade6B), imr1R(Sn:ade6X), rad51::kanMX6, pof3-L148R*
TNF4119	*h^+, ade6Δ-D, imr1L(Sn:ade6B), imr1R(Sn:ade6X), rad54::kanMX6, pof3-L148R*
TNF4111	*h^+, ade6Δ-D, imr1L(Sn:ade6B), imr1R(Sn:ade6X), rad52::kanMX6, pof3-L148R*
TNF5999	*h^+, ade6Δ-D, imr1L(Sn:ade6B), imr1R(Sn:ade6X), rad52-R45K*
TNF6019	*h^+, ade6Δ-D, imr1L(Sn:ade6B), imr1R(Sn:ade6X), rad51::kanMX6, rad52-R45K*
TNF6009	*h^+, ade6Δ-D, imr1L(Sn:ade6B), imr1R(Sn:ade6X), rad52-R45K, pol1-R961K*
TNF6037	*h^+, ade6Δ-D, imr1L(Sn:ade6B), imr1R(Sn:ade6X), rad51::kanMX6, rad52-R45K, pol1-R961K*
TNF3710	*h^+, ade6Δ-D, imr1L(Sn:ade6B), imr1R(Sn:ade6X), swi6::hphMX6*
TNF4542	*h^+, ade6Δ-D, imr1L(Sn:ade6B), imr1R(Sn:ade6X), rad51::kanMX6, swi6::hphMX6*
TNF6655	*h^+, ade6Δ-D, imr1L(Sn:ade6B), imr1R(Sn:ade6X), rad52::kanMX6, swi6::hphMX6*

TNF5096	h^+, ade6Δ-D, imr1L(Sn:ade6B), imr1R(Sn:ade6X), cdc18-K46
TNF5155	h^+, ade6Δ-D, imr1L(Sn:ade6B), imr1R(Sn:ade6X), rad51::kanMX6, cdc18-K46
TNF6632	h^+, ade6Δ-D, imr1L(Sn:ade6B), imr1R(Sn:ade6X), rad52::kanMX6, cdc18-K46
TNF4594	h^+, ade6Δ-D, imr1L(Sn:ade6B), imr1R(Sn:ade6X), cdc20-M10
TNF4617	h^+, ade6Δ-D, imr1L(Sn:ade6B), imr1R(Sn:ade6X), rad51::kanMX6, cdc20-M10
TNF5037	h^+, ade6Δ-D, imr1L(Sn:ade6B), imr1R(Sn:ade6X), rad52::kanMX6, cdc20-M10
TNF5018	h^+, ade6Δ-D, imr1L(Sn:ade6B), imr1R(Sn:ade6X), swi1::hphMX6
TNF5033	h^+, ade6Δ-D, imr1L(Sn:ade6B), imr1R(Sn:ade6X), rad51::kanMX6, swi1::hphMX6
TNF6653	h^+, ade6Δ-D, imr1L(Sn:ade6B), imr1R(Sn:ade6X), rad52::kanMX6, swi1::hphMX6
TNF4328	h^+, ade6Δ-D, imr1L(Sn:ade6B), imr1R(Sn:ade6X), swi7-1
TNF3983	h^-, smt0, leu1-32, ade6Δ-D, imr1L(Sn:ade6B), imr1R(Sn:ade6X), rad54::kanMX6
TNF4125	h^-, smt0, leu1-32, ade6Δ-D, imr1L(Sn:ade6B), imr1R(Sn:ade6X), rad54::kanMX6, pol1-R961K
TNF4109	h^-, smt0, leu1-32, ade6Δ-D, imr1L(Sn:ade6B), imr1R(Sn:ade6X), rad54::kanMX6, spb70-G529D
TNF4102	h^-, smt0, leu1-32, ade6Δ-D, imr1L(Sn:ade6B), imr1R(Sn:ade6X), rad54::kanMX6, pof3-L148R
TNF2648	h^+, ura4-DS/E, otr1R(SphI)-$ura4^+$
TNF4208	h^+, ura4-DS/E, otr1R(SphI)-$ura4^+$, pol1-R961K
TNF4625	h^+, ura4-DS/E, otr1R(SphI)-$ura4^+$, swi7-1
TNF3220	h^+, ura4-DS/E, otr1R(SphI)-$ura4^+$, swi6::hphMX6
TNF35	h^+
TNF2134	h^+, rad52-6His3Flag:kanMX6
TNF3696	h^+, rad52::kanMX6
TNF5492	h^+, rpa2-mCherry:hphMX6
TNF5511	h^+, rpa2-mCherry:hphMX6, pol1-R961K

Supplementary Table 2. DNA primers used in this study.

Primer	Sequence
cn1	5′–AACCGCAACAAACGATTAGC–3'
cn2	5’–CGGAATTAGAAAGATTGATGATTTG–3'
im1	5’–AAGTTTTGATGCTCAACAAATGGC–3'
rc1	5’–CATTAAAAATCAACAAGTCTTGTCC–3'
rc2	5’–GTTACTATGGATAAAGATAATTGTTT–3'
rad52-N-F1	5'–CGCAAAGCAGATTGCAAAGG–3'
rad52-RK-R	5'–GGACCTGACTTTCTTGAAACG–3'
rad52-RK-F	5'–CGTTTCAAGAAAGTCAGGTCC–3'
rad52-C-R	5'–CCTCTGCTACTGCTAAATGAGC–3'
