# Supplementary material for: DNA replication machinery prevents Rad52-dependent single-strand annealing that leads to gross chromosomal rearrangements at centromeres
Source: Commun Biol. 2020 Apr 30;3:202. doi: 10.1038/s42003-020-0934-0 (PMC7193609; doi:10.1038/s42003-020-0934-0)
Supplement: Supplementary file 2 — Description of Additional Supplementary Files [file 42003_2020_934_MOESM2_ESM.pdf]

Description of Additional Supplementary Files

File Name: **Supplementary Data 1**
Description: source data for Figs. 1c, 1d, 2d, 3b, 3d, 4c, 4f, 5a, 5b, 5c, 6d, 6e, 6f, 6g, 6h, 6i, 7a, 7b, and Supplementary Figs. 3c, 4b, and 6b on separate sheets.
